# *Bróderes* in arms: Gangs and the socialization of violence in Nicaragua

**DOI:** 10.1177/0022343317714299

**Published:** 2017-09-12

**Authors:** Dennis Rodgers

**Affiliations:** University of Amsterdam

**Keywords:** gangs, Nicaragua, socialization, violence

## Abstract

Drawing on longitudinal ethnographic research that has been ongoing since 1996, this article explores the way that gangs socialize individuals into violent norms and practices in Nicaragua. It shows how different types of gang violence can be related to distinct socialization processes and mechanisms, tracing how these dynamically articulate individual agency, group dynamics and contextual circumstances, albeit in ways that change over time. As such, the article highlights how gang socialization is not only a variable multilayered process, but also a very volatile one, which suggests that the socialization of violence and its consequences are not necessarily enduring.

## Introduction

Youth gangs are one of a small number of truly global social phenomena, present across time and space in almost every society on the planet (see Hazen & Rodgers, 2014). Although significant variation can be observed between different contexts, a universal feature of gangs is their intimate association with violence. At the same time, as Stretesky & Pogrebin (2007: 85) have remarked, ‘few studies have examined how violent norms are transmitted in street gangs’. Most of the scholarly literature generally tends to assume that youth who join gangs are either violent by nature or due to factors associated with their social environment, or else that they will be institutionally ‘socialized’ into diverse practices of violence by the gang. As Checkel (2017) points out in the introduction to this special issue, however, socialization – that is to say, the means through which individual actors adopt particular norms, rules, and practices associated with membership of a given group – is neither obvious nor predetermined, and may be underpinned by a range of different processes and mechanisms.

Understanding how these operate and interact is obviously critical for coherent violence reduction and peacebuilding. Drawing on longitudinal ethnographic research carried out in the poor neighbourhood *barrio* Luis Fanor Hernández,^1^ in Managua, the capital city of Nicaragua, this article therefore explores how local gang members have assimilated and put into practice a range of different forms of violence over the past quarter century, unpacking the way these processes have changed over time, and highlighting how socialization is a fundamentally multilayered and contingent process. It begins by situating gangs and their violence within the Central American and Nicaraguan contexts. It subsequently offers an overview of theories of gang socialization, before arguing for a tripartite approach focusing on the variable configuration of individual agency, group dynamics and contextual circumstances. This is then empirically illustrated in relation to a range of distinct forms of violence associated with different iterations of the *barrio* Luis Fanor Hernández gang, before a conclusion offers some general reflections and an agenda for future research.

## Gangs in Central America and Nicaragua

Although gangs are a global phenomenon, nowhere are they currently more the focus of attention than in contemporary Central America, where they are widely reported to be among the most important actors in a landscape of criminal brutality characterized by levels of violence that often surpass those that afflicted the region during the revolutionary wars and conflicts of the 1970s and 1980s (see Kruijt & Koonings, 1999). At the same time, estimates of the total proportion of contemporary regional violence attributable to gangs vary wildly from 10% to 60% (UNODC, 2007: 64), while the criminal activities with which they are associated range from localized forms of petty delinquency such as theft, muggings and extortion, to more large-scale organized criminality including migrant trafficking, kidnapping and drug trafficking. This uncertainty is further fuelled by the fact that Central American gangs have been the focus of a steady stream of highly sensationalist publications over the past decade, for example speculating that gangs constitute ‘a new urban insurgency’ (Manwaring, 2005).

As Hagedorn (2008) has remarked, ‘branding gangs a “national security threat” […] is consistent with a[n] […] attitude that divides the world into good and evil’, thereby establishing the basis for highly distorted interpretations. For example, there exists significant diversity both between gangs and between countries within the region. Certainly, a critical distinction must be made between *pandillas*, on the one hand, and *maras*, on the other. *Maras* are a phenomenon with transnational roots, linked to the refugee flows from Central America to the USA in the 1980s and the subsequent deportation of refugee youth exposed to US gang culture in the 1990s (see Valdez, 2011). *Pandillas*, on the other hand, are more localized, home-grown gangs that are the direct inheritors of the youth gangs that have long been a historic feature of Central American societies. *Maras* are generally associated with much more intense and spectacular forms of violence than *pandillas*, partly because they are less embedded within their local institutional context than the latter, and are therefore less rule-bound and constrained (see Rodgers & Baird, 2015: 479–483).^2^

*Pandillas* are only significantly visible in Nicaragua, having been almost completely supplanted by *maras* in El Salvador, Honduras and Guatemala during the post-Cold War period;^3^ this is one reason why Nicaragua suffers less violence than the latter three countries.^4^ At the same time, even if they are less violent than *maras*, *pandillas* remain a major source of insecurity in Nicaragua. Certainly, this is something that has been highlighted by numerous ethnographic investigations over the past two decades (see e.g. Núñez, 1996; Rocha, 2007, 2013; Rodgers, 2000, 2006, 2007a,b, 2014, 2015, 2016; Weegels, 2017), as well as opinion polls. A 1999 survey conducted by the Nicaraguan NGO Ética y Transparencia, for example, found that 50% of respondents identified gangs as the principal threat to their personal security (Cajina, 2000: 177). More than a decade later, the 2011 Citizen Security Perception Survey carried out by the Managua-based Institute for Strategic Studies and Public Policy found that almost 60% of respondents considered gangs the most important security threat in Nicaragua (Orozco, 2012: 8).

*Pandillas* are not a new feature of Nicaraguan society, and can be traced back to the 1940s and 1950s, when the country urbanized on a large scale. These first gangs were spontaneous groups of youths that emerged organically in urban slums and squatter settlements, and only lasted so long as the specific peer group underpinning them stayed together. Gangs subsequently declined during the 1970s as a consequence of the intensification of the long-standing *Sandinista* revolutionary struggle against the Somoza dictatorship, and disappeared almost completely following the triumph of the revolution in 1979 due to the introduction of universal military service – the age of conscription being 16 – and also because of the grassroots organization that was a hallmark of *Sandinismo*, including youth work brigades and extensive local neighbourhood watches. Gangs, however, began to re-emerge during the late 1980s due to the war-fuelled erosion of the *Sandinista* welfare state, declining levels of local organization, the decreasing legitimacy of the revolutionary regime and increasing numbers of youths deserting their military service (Lancaster, 1992: 132).

These new gangs principally involved groups of young men^5^ who had been conscripted together and who joined forces in order to protect their families and friends from the rising crime and insecurity, thereby displaying something of a vigilante ethos. Following regime change in 1990, gangs began to proliferate exponentially as a result of peace and mass demobilization, becoming a ubiquitous feature of poor urban neighbourhoods in the country’s major cities (see e.g. Núñez, 1996; Rodgers, 2000). By the mid-1990s, a full-blown gang culture had institutionalized, with gang members engaged in a wide range of petty delinquency, while rival gangs collectively fought each other for territorial control. These conflicts principally revolved around protecting local neighbourhood inhabitants from rival gangs; due to their fixed nature and their adherence to processes of regular escalation, they provided a measure of predictability within an otherwise chaotic and highly insecure broader social context. In that sense, the original vigilante ethos of the first postwar generation persisted, despite individual turnover due to gang members ‘maturing out’ between the ages of 19 and 22 (Rocha, 2000a,b; Rodgers, 2006, 2007a).

Gangs changed radically around the turn of the century, however. In particular, they shifted from offering localized forms of protection and social order to being much more parochial, predatory and feared organizations. This shift was largely linked to the spread of cocaine in Nicaragua. The drug began to move through the country in substantial quantities from 1999 onwards,^6^ and its consumption in the form of crack rapidly became a major element of gang culture. Although gang members in the early and mid-1990s consumed drugs, cocaine was practically unknown at that time and they mainly smoked marijuana or sniffed glue. Unlike the latter, crack makes its users extremely aggressive, violent and unpredictable; its consumption thus led to a rise in spontaneous, random attacks by addicted gang members looking to obtain money for their next fix. Contrary to the past, gang members began to actively target local residents, generating a widespread and tangibly heightened sense of fear in urban neighbourhoods in Managua and other Nicaraguan cities, from around 2000 onwards. In other words, crack consumption fundamentally changed the nature of the relationship between gangs and their local communities (Rocha, 2007).

In some neighbourhoods, gang members integrated into the emergent Nicaraguan drug economy as street dealers, further increasing insecurity in those areas. For the most part, dealers worked independently, selling irregularly on street corners in their neighbourhood and sourcing their crack cocaine from one of a small number of neighbourhoods in the city, where it was initially distributed by individual distributors on a rather ad hoc basis (see Rodgers, 2016). These were often ex-gang members who drew on their historical links to their local gang to enrol current members as their security apparatus. In these neighbourhoods, gang activities shifted from community protection to ensuring the proper functioning of the drug economy, which they achieved by imposing local regimes of terror that went beyond the more diffuse crack consumption-related violence. To reduce the risk of denunciation, gang members fostered a climate of fear by repeatedly threatening and committing arbitrary acts of violence against local community inhabitants (Rodgers, 2006, 2007b; Rocha, 2007).

From the beginning of the 21st century – but most visibly around 2005 – the number of gangs in Nicaraguan cities began to decline (Rocha, 2007). The trend was attributable partly to the atomizing effect of crack consumption (see Rodgers, 2006), and partly to the emergence of more professional drug-dealing groups, often referred to as *cartelitos* (little cartels). These groups generally involved individuals from several different neighbourhoods, and even different parts of Nicaragua, and brutally repressed local gangs to prevent them from challenging them (Rodgers, 2015). This violence reached a peak around 2009–10, after which it eased up significantly as *cartelitos* began to either fall apart due to internecine fighting or were taken over by rivals. Those that remained began to reduce their involvement in local drug dealing and refocused on drug trafficking, which has much higher profit margins. Instead of dominating local communities, *cartelito* members began to minimize their visibility, which led to improvements in local security in the urban neighbourhoods where they had previously operated. While drug dealing continues to be widespread in Nicaraguan cities, it has become much smaller in scale, disorganized and more individualized. Gangs, for their part, continue to be a feature of many poor urban neighbourhoods in present-day Nicaragua, but not to the same degree as during the 1990s and the early years of the following decade (see Rodgers & Rocha, 2013: 58–59).

## Theorizing gang socialization

Broadly speaking, there exist three major approaches to explaining gang violence within the scholarly literature. The first sees it as linked to the individual personality traits of gang members. This encompasses approaches that see gang members as psychopaths – see Yablonsky (1963), for example – to more nuanced analyses that consider them to be representative of specific psycho-social personalities, such as ‘defiant individualism’ (Sánchez Jankowski, 1991; see also Bernard, 1990). This conception of gang violence is rather self-serving, and no investigation has convincingly shown that gang members consistently display any particular personality type (see Covey, 2003; Curry, Decker & Pyrooz, 2014: 38–42; Klein & Maxson, 2006). Moreover, for every gang member who might plausibly be categorized as a psychopath or a ‘defiant individual’, there are generally at least an equal if not a greater number of non-gang members displaying the same personality type within any given context (as well as gang members who are not ‘defiant individualists’). Having said this, while violence cannot be said to be an innate trait of gang members, numerous studies have highlighted the importance that specific individuals can have in relation to the socialization of violence within a gang, whether as influential leaders (Whyte, 1943; Decker & van Winkle, 1996), or as providers of specialized expertise (Keiser, 1969; Rodgers, 2016), although as Fischer (1975) points out, individuals by themselves do not make a gang, and there clearly needs to be a demographic ‘critical mass’.

The second major way in which gang violence has been understood within the scholarly literature is as a corollary of group dynamics. This makes sense from a socialization perspective considering that at its most basic it involves a process whereby an individual is assimilated into a collective. The gang group is in other words a source of socialization, shaping members’ sense of self and identity, including the internalization of specific norms and practices. The question, however, is how this actually takes place beyond simply joining the gang. In his foundational study of early 20th-century Chicago gangs, Thrasher (1927: 29–30) famously argued that their violence was the result of ‘spontaneous play-groups’ acquiring ‘group-consciousness’ through ‘opposition’ to ‘a rival or an enemy’, a process that effectively amounts to the institutionalization of gang dynamics through conflict. Such an assertion has been widely repeated by gang scholars in numerous contexts over the years – see Suttles (1968), Lepoutre (1997), or Jensen (2008), for instance – yet Thrasher’s observation arguably applies to any competitive sports team, and he never explains what it is about ‘opposition’ that institutionalizes gangs as violent organizations. The notion of ‘spontaneous’ group formation is moreover similarly glossed over, except to the extent that Thrasher relates gang formation to race and ethnicity, effectively suggesting that they emerge based on pre-existing group dynamics.

The third major approach to explaining gang violence is contextually. Certainly, there exists a long tradition associating gangs with ‘social disorganization’ (Thrasher, 1927), or in other words, as social consequences of poverty and marginality. This correlation derives from an epistemological conception that sees youth socialization as normally occurring via a range of ‘primary’ social institutions such as families and schools, which when absent or deficient (due to poverty and marginality), are replaced organically by more ‘secondary’ – and frequently dysfunctional – local social institutions such as gangs. According to Vigil (1988, 2002), however, gangs provide their members with a form of ‘street socialization’ that has been particularly well described by Anderson (1999) in his famous study of the ‘code of the streets’ characteristic of poor inner-city black neighbourhoods in Philadelphia. Due to the pervasive scarcity and increased competition resulting from poverty and discrimination in these communities, Anderson (1999: 32–33) argues that ‘an oppositional culture’ based on ‘the use of violence’ emerges, ‘at the heart of [which] is the issue of respect – loosely defined as being treated “right” or being granted one’s “props” (or proper due) or the deference one deserves’. This most evidently shapes interactions on the streets and other public spaces in poor neighbourhoods, and as one of the major social institutions physically occupying the streets in such contexts, gangs effectively come to crystallize a heightened expression of this ‘code’, which their members adopt as their principal way of being.

While there is no doubt that gangs are generally street-based organizations, and that they can be linked to broader structural processes such as poverty and marginalization, generally invoking contextual factors to account for their violence fails to explain why only a minority of youth – generally less than 10% within any given context (Vigil, 1988: 422) – ever join a gang and become regularly involved in violence. Contextual circumstances by their very nature impact on all those living within a given context; indeed, Anderson’s (1999) study of the ‘code of the street’ repeatedly illustrates that it is a way of acting that is not specific to gangs. To this extent, the idea of ‘street socialization’ can be said to implicitly associate violence with gang membership in a rather singular manner. While the notion that material circumstances might lead to processes of socialization of particular norms and practices is by no means unreasonable, these will clearly be mediated by other factors, including most obviously more individual and group-based forms of socialization. Having said this, there clearly also exists something of a feedback mechanism here; as Ayling (2011) has pointed out, gangs are generally highly volatile social institutions, and their internal dynamics are particularly susceptible to changing contextual factors (see also Sánchez Jankowski, 2003).

Ultimately, what these different ideas about gang socialization arguably implicitly highlight is that socialization must be conceived as a multifaceted process rather than a singular event, and that it can potentially involve a range of different factors, including in particular individual agency, group dynamics and contextual circumstances. These can, however, clearly play out in variable ways; there are, for example, differences between the effects of pre-existing, institutionalized and organic group dynamics, while different types of contextual constraints impact on individual and endogenous group logics in a similarly mutable manner. At the same time, the existing scholarly literature also suggests that such patterns are likely to be highly variable across time and space – meaning that trying to determine whether one factor is more important than another is very likely a fruitless exercise – and what is perhaps most important and interesting to understand instead is how and why individual agency, group dynamics and contextual circumstances connect and articulate together in order to produce specific socialization configurations, practices and outcomes.

In other words, it is not only important to identify how different forms of socialization can be associated with distinct forms of violence, but also how different iterations of individual agency, group dynamics and contextual circumstances can combine with each other in order to produce particular outcomes. It is critical that this is unpacked to understand whether there exist socialization configurations that lead to more or less – as well as more durable or more volatile – patterns of violence. The next section of this article therefore draws on the empirical example of the evolution of a specific gang in a poor Managua neighbourhood called *barrio* Luis Fanor Hernández to explore how such a tripartite and interconnected conception of socialization might allow for a better understanding of the dynamics of gang violence.^7^ More specifically, it seeks to trace the changing patterns of violence associated with different temporal iterations of the local gang, and relate their socialization to distinct configurations of individual agency, group dynamics and contextual circumstances. It aims to identify how these different elements connected to each other, as well as chart the way their relationship transformed over time, focusing on three specific issues, namely whether the connection between different forms of socialization is systemic or contingent, whether any form or socialization configuration is more durable than another, and finally, whether the form of violence being socialized makes a difference in the process of socialization.

## Gang socialization in *barrio* Luis Fanor Hernández

*Barrio* Luis Fanor Hernández is a poor urban neighbourhood located in southeast Managua, the capital city of Nicaragua. The locality was originally founded as an illegal squatter community by rural-urban migrants in the early 1960s, one of many such informal settlements that mushroomed on the edge of Managua at that time. Due to its inhabitants’ extreme poverty, the settlement was initially known as *La Sobrevivencia* (Survival), but was completely rebuilt during the early 1980s as a beneficiary of the revolutionary *Sandinista* state’s housing development programme, and renamed *barrio* Luis Fanor Hernández (after a local ‘martyr of the Revolution’), although socio-economically it remained in the lowest quartile of Managua neighbourhoods. The settlement has always been infamous for its high levels of criminality, but became extremely notorious in the early 1990s due to the emergence of a very brutal local gang. This bad reputation has persisted into the present, although the gang has changed significantly over the past two decades, even disappearing completely for a few years during the latter half of the 2000s.

The evolution of the *barrio* Luis Fanor Hernández gang since 1990 has largely corresponded to the broader evolution of Nicaraguan gangs described above, and can be divided into five phases, each associated with different forms of violence and different socialization mechanisms, as summarized in Table I. Due to space considerations, rather than offering a systematic chronological overview of different phases, this section moves between specific examples of distinct forms of socialization, tracing how

**Table I.** Gang evolution in *barrio* Luis Fanor Hernández

*Historical gang phase*	*Period*	*Size of the gang*	*Type of violence associated with the gang*	*Key socialization mechanisms*
Emergent	1989–92	14	Vigilantism; knowledgeable use of firearms	General contextual factors; pre-existing group dynamics
Golden era	1993–98	c. 100^a^	Ritualized intergang warfare; variable use of firearms	Local contextual factors; institutionalized group dynamics; individual agency
Drug dealing	1999–2005	18–20	Instrumental violence to support local drug economy; knowledgeable use of firearms	Activity-related contextual factors; individual agency
Pacification	2006–11	N/A	N/A	N/A
Revival	2012–	12	Spontaneous intergang conflicts; unknowledgeable use of firearms	Organic group dynamics

^a^The element of imprecision regarding this figure is due to the fact that the gang included both ‘core’ and ‘peripheral’ members during this phase, and the composition of the peripheral group was very fluid.

they interrelated to each other, how they changed over time, and what consequences this change had for the institutionalization of violence in the gang. It should be noted that the socio-economic background of gang members has generally not changed significantly across these five different phases (see Rodgers, 2014), and nor has their gender, as all *barrio* Luis Fanor Hernández gang members since 1990 have been young men, with one exception.^8^ On the other hand, the gang’s structure has evolved over time, both in terms of its size and organization, insofar as it has been hierarchical and stratified as well as egalitarian and amorphous at different points in time. Both the spread and median age of members have also fluctuated across phases, with spread ranging from 7 to 26 years of age, while the median has varied between 15 and 24 years of age.

The different phases of the gang’s evolution, and in particular its changing patterns of violence, can clearly be related to distinct processes and mechanisms of gang socialization encompassing individual agency, different types of group dynamics and varyingly influential contextual factors, as Table I highlights. For example, the vigilante violence of the first post-1990 iteration of the *barrio* Luis Fanor Hernández gang was clearly directly connected to the fact that most of its members were demobilized *Sandinista* Popular Army conscripts. The core of the gang was a group of eight youths aged between 18 and 20 years old who were demobilized more or less simultaneously in 1989. They began to hang out together on a neighbourhood street corner, along with four slightly older youths aged between 20 and 23 years old, three of whom had also been conscripts, as well as two younger individuals aged respectively nine and ten years old who gravitated to the group for idiosyncratic reasons (see Rodgers, 2016). This group was quickly labelled a ‘*pandilla*’, both by its members and inhabitants of *barrio* Luis Fanor Hernández more generally, particular once the members of the group began to engage regularly in a range of violent activities. Most of the time these involved beating up individuals who had robbed, attacked or threatened the friends or family of gang members, something that happened frequently in the post-war context of heightened flux and uncertainty that characterized Nicaragua in the early 1990s.

The impulse for this particular pattern of violence was clearly related to the ex-conscript nature of gang members. Certainly, all the members of the gang from this period whom I interviewed systematically highlighted three basic reasons for forming a gang and acting as they did, all of which were directly related to their ideological experiences as conscripts. Firstly, the change of regime in 1990 had led to an abrupt reduction of their social status. Their role as conscripts ‘defending the nation’ and ‘the Revolution’ had previously been held in very high esteem in their community, and forming a gang and being violent had offered them a means of reaffirming their status vis-à-vis a wider society that seemed to forget them very rapidly in the post-conflict period. Secondly, becoming gang members had been a way for them to recapture some of the adrenaline-charged energy of war, while also reconstituting a comradeship and solidarity reminiscent of their wartime experiences as conscripts. But perhaps most importantly, they saw becoming gang members as a natural continuation of their previous role as soldiers. The early 1990s had been highly uncertain times, marked by political polarization, violence and spiralling insecurity, and these youths felt they could better ‘serve’ their families and friends by joining a gang than attempting to ‘protect’ them as individuals (see Rodgers, 2006: 283–284). From a socialization perspective, then, the first generation of post-conflict *barrio* Luis Fanor Hernández gang members had clearly been collectively ‘pre-socialized’ into their distinct pattern of violence due to the group’s experience of conscription as well as, more indirectly, general contextual factors in the form of their general experience of *Sandinista* revolutionary ideology. This highlights very well the interrelation between group dynamics and contextual circumstances, which also included more contingent ones in the form of the local ambient chronic insecurity.

From 1992 onwards, however, the ex-conscript members of the first postwar iteration of the *barrio* Luis Fanor Hernández gang began to ‘mature out’ of the gang.^9^ They were replaced by new members who had no military background or significant experiences of *Sandinismo*, and moreover after 1994 no direct link to the first generation of ex-conscript *barrio* Luis Fanor Hernández gang members. Yet, the vigilante norms and practices of previous gang members continued to influence new members due to a transformation in the way that contextual factors such as *Sandinista* revolutionary ideology were internalized by gang members. Rather than being based on gang members’ ideological experience of ‘defending the Nation’ and ‘the Revolution’, the *barrio* Luis Fanor Hernández gang’s vigilante impulse became linked to a local contextual sense of belonging to the neighbourhood, as became clear during a conversation I had one morning in October 1996 with a gang member called Julio. I came across him as he was cleaning up graffiti from the 1980s, which extolled the virtues of the *Sandinista* youth organization; a person or persons unknown had crudely painted it over in bright red – the colours of the anti-*Sandinista* Constitutionalist Liberal Party – the night before. As Julio angrily berated the ‘*hijos de la setenta mil putas*’ (‘sons of seventy thousand whores’) who had done this, I initially assumed that this was an exemplification of his *Sandinista* sympathies, but it quickly became apparent that he saw this act of vandalism less as an attack on *Sandinismo* and more as a desecration of a material manifestation of *barrio* Luis Fanor Hernández’s identity:

Those *jodidos* [assholes] don’t respect anything in the neighbourhood, Dennis, nothing! OK, so they don’t like *Sandinismo*, that’s how it is, I don’t like their politics either, but this is more than just a *Sandinista pinta* [graffiti], it’s a part of the neighbourhood history. *Our* history, *bróder*! It’s something that belongs to the community, to all of us; it shows us who we are, where we come from, how *Sandinismo* built our houses and made us into a community. It shows what the neighbourhood is, and people should therefore respect it, whatever their political opinions.

This particular discourse clearly highlights how Julio’s putative *Sandinista* sympathies actually derived from a sense of territorial identity rather than politics. In broader sociological terms, this can be linked to the shrinking of the collective social imaginary in post-revolutionary Nicaragua that Núñez (1996) has described as involving an ontological shift ‘from the nation to the neighbourhood’. In relation to gang socialization, however, what this allowed for was a continuation of the conscript-derived vigilante ethos of the first iteration of the *barrio* Luis Fanor Hernández gang, albeit in a transformed manner. Rather than deriving from general ideological considerations, this became linked to a specific form of local territoriality. This process of territorialization also affected the violent practices of the gang in a more practical way. While the first gang’s vigilante violence had been rather ad hoc in nature, and principally aimed against individuals perceived as threatening, the *barrio* Luis Fanor Hernández gang’s mid-1990s iteration displayed more territorial dynamics. Its violence now revolved around semi-ritualized forms of gang warfare that rigidly obeyed a number of precise rules and practices and involved either attacking or protecting a neighbourhood to engage enemy gangs, with fighting generally specifically focused either on harming or limiting damage to both neighbourhood infrastructure and inhabitants, while injuring or killing symbolically important enemy gang members (for more details see Rodgers, 2006).

Gang warfare was in and of itself clearly constitutive of both the gang group and individual gang members, as the latter were collectively socialized through combat, learning to fight for and with each other, as well as from each other, with younger gang members in particular learning from the actions of older ones. This meant that rather than deriving from a ‘pre-socialized’ group sharing a life trajectory of conscription, the gang’s violence became institutionalized into the group’s dynamics, and it was not necessary for new members to have shared prior experiences in the same way that the first wave of conscript-gang members had. To this extent, gang violence responded to a different socialization configuration, one that still combined group dynamics – but newly institutionalized rather than pre-existing – and contextual circumstances – but purely local rather than ideological.

At the same time, the gang’s violent practices also owed much to the fact that the two younger members of the first iteration of the *barrio* Luis Fanor Hernández gang had remained as members of its second incarnation. In particular, Milton and Bismarck played an active role in facilitating the practical transmission of certain types of violent practices that were directly linked to the conscript experiences of the first postwar generation of *barrio* Luis Fanor Hernández gang members, including more specifically those concerning the use of firearms. Guns have of course long been associated with gangs; as Arendt (1969: 4) famously pointed out, violence ‘always needs implements’. The use of firearms, however, requires specialized knowledge; guns are by no means intuitive, as a former gang member called Jorge highlighted during the course of an interview in 2012, when he recounted the first time that he had tried to use a gun in the early 1990s:

I was 13–14 years old […] The gun was my father’s, he’d brought it back from military service after the war. He kept it locked in a drawer, but whenever he’d get drunk, he’d take it out, and wave it at the neighbours, pretending to shoot them. One day, I broke into the drawer and took the gun, you know, to go and mug somebody. I’d been hanging around with the gang, you see, and the day before one of them got a really nice pair of Nike shoes by pulling a gun on some rich kid, and I thought ‘why don’t I get myself some nice shoes too’, and so went to the *Colonia* Las Condes with the gun to find somebody to hold up. It didn’t go as planned, though, as the guy I tried to rob refused to give up his shoes. When I tried to shoot him, nothing happened, because the safety catch was on! I was so dumb, I didn’t know that guns had safety catches then, and so I just dropped the gun and ran away, because he was much bigger than me. I can laugh about it now, but I was scared shitless […] You know what the worse thing was, though? I lost the gun, and so my father really beat me up afterwards […]

The first generation of gang members in *barrio* Luis Fanor Hernández either obtained their specialized knowledge about guns directly, during their military service, or they were taught by a gang member who had done military service. As Bismarck, for example, who had no military experience, put it in an interview in 2012: ‘We were taught how to use firearms by the gang members who had done their military service […] They showed us how to load guns, how to shoot them, how to strip and clean them.’ Although the ex-conscript members of the first *barrio* Luis Fanor Hernández gang had all matured out by the mid-1990s, the fact that Bismarck and Milton continued on as gang members meant that they acted as bridges for the transmission of this specialized knowledge, along with more general notions about fighting, actively socializing the new generation of gang members into practices of violence, as the following extract from the 4 December 1996 entry in my field diary illustrates well:

Today I was interviewing Milton when he suddenly interrupted our conversation to summon over Chucki, who was passing by. ‘*Oye* Chucki,’ he shouted, ‘*venivé*, I want to show you something.’ Chucki duly sauntered up to us, only to be knocked hard to the ground when Milton sucker-punched him in the balls. Writhing in pain, Chucki screamed ‘why the fuck did you do that, *hijuéputa*?’, to which Milton coolly replied, ‘because you’ve got to learn, *maje*, you’re new to the gang and you don’t know anything yet.’ He then turned to me, and said, ‘*ves*, Dennis, that’s how you teach the young ones. They’ve got to learn how to take it and to be prepared for anything. Otherwise they don’t last long. That’s how I learnt after I joined the gang, from the older *bróderes* (brothers) – they taught me how to fight, how to defend myself, all that kind of stuff […] Now that I’m one of the older guys and I know what I’m doing, it’s my job to teach the new guys […]

Although this kind of behaviour obviously also played a role in confirming gang hierarchy, it constituted an individualized form of socialization into violence, dependent on the acts of specific actors. At the same time, although such processes of socialization continued across successive generations of gang members, they were clearly more volatile than either group dynamics or contextual circumstances. For example, as the temporal distance from the generation that had had professional or near-professional training increased, there was something of a ‘Chinese whispers’^10^ effect with regards to firearms use. This became acute after Milton and Bismarck both ‘retired’ from the gang, respectively in 1997 and 1999, which meant that knowledge about guns in the late 1990s began to be acquired third or fourth hand by new gang members. This affected its quality, as was apparent from the way that the number of gang members suffering accidents involving firearms soared in the late 1990s compared to the early 1990s and mid-1990s.

Indeed, in an interview in 2002, Bismarck explicitly linked the rising number of accidents to weapons becoming increasingly defective due to lack of care, as well as the fact that gang members did not always understand how to use their weapons, often unintentionally shooting themselves or others. ‘Gang members nowadays don’t take proper care of their weapons, so they’re breaking down all the time, sometimes even blowing up in their face,’ he told me, before then going on to discuss the case of a young gang member who had recently shot himself in the foot:

He had no idea what he was doing. He’d got this pistol, and thought that made him a *poderoso* (big man), but you know, you’ve got to know how to use a gun to be able to do something with it. He shot himself because he put it in his belt without the security turned on […] The problem was that he hadn’t had proper training, because there’s nobody left in the gang who really knows, and so he’d only half understood things, or hadn’t been told properly, and that’s why he shot himself.

Partly because of this, the levels and intensity of the *barrio* Luis Fanor Hernández gang’s violence declined during the late 1990s, pointing to the contingent importance of individual agency in gang socialization. This was also evident in the fact that there was a renewal of gang member knowledge about firearms in *barrio* Luis Fanor Hernández in the early 2000s, due to an ex-gang member from the mid-1990s called Jhon, who spent five years in the Nicaraguan Army. He had joined the neighbourhood gang in 1994 at the age of 13 but was sent to the Army by his family in 1997 because they could ‘no longer cope with him’ and hoped that it would ‘educate him’, as his mother *Doña* Aurora put it in an interview in 2007. After he returned to the neighbourhood in 2002, Jhon re-joined the gang and his expertise in weapons critically transformed the levels of gang member knowledge about guns, as he explained during the course of an interview in 2012:

[The Army is] where I learnt to use firearms, the AK-47, the sniper rifle, the RPG – which is a rocket-launcher – all kinds of weapons! I had classes, it was like school, and they taught us to shoot, to strip and clean our weapons, and there were also exams. I can strip and re-assemble any kind of weapon – I know everything, I tell you! The basic weapon in the Army was the AK-47, but because I could shoot really well, I became a sniper, and so used a special rifle. I went and trained in Martinique and Marie-Galante, they’re French islands, and I trained with the French Army and also the Venezuelan Army […] All of this helped me when I came back to the neighbourhood afterwards […] During my service I’d come back every 15 days, and whenever I came, all the *bróderes* would say, ‘bring me a gun, *mon*, bring me an AK’, but I’d just say to them, ‘*oye maje*, do you know how to use a gun?’ I’d tell them that I wasn’t going to bring anything if they didn’t know how to take care of their guns, if they couldn’t strip and re-assemble them. I told them that they needed to learn all of this, and so they asked me to teach them. So after a while, I brought back an AK-47 and taught them all, in groups of five […] You see, an AK-47 isn’t complicated, but there’s a specific order you have to follow to strip it in order to be able to clean it. The first thing you do is release the magazine catch, then you remove the magazine, then you cock the rifle, and – then – you take off the receiver cover and the recoil mechanism […] Then you remove the bolt carrier and then the bolt, and then you release the catch on the right side of the rear sight, and take off the hand guard, and then all that’s left is the skeleton, which you clean. Afterwards, to re-assemble it, you just put everything back together in the reverse order.

Between 2002 and 2005, the *barrio* Luis Fanor Hernández gang became one of the most feared gangs in the southeast of Managua due to its effective deployment of firearms because of Jhon’s training, highlighting how a gang’s trajectory of violence can change for very contingent – and individual – reasons. At the same time, other factors also affected the gang’s violence during this period. These were linked to the development of a local drug economy in *barrio* Luis Fanor Hernández, which emerged in 1999–2000. This initially began very much in an ad hoc manner, centred on a single individual who was a former gang member from the early 1990s, and who rapidly recruited current gang members to act as his street dealers. The gang also acted collectively as the nascent drug economy’s security apparatus, enforcing contracts and guarding drug shipments whenever they entered or left the neighbourhood, as well as engaging in a campaign of predatory terror to intimidate local inhabitants, arbitrarily threatening, beating and intimidating to prevent denunciations, and to ensure that drug dealing in the neighbourhood could occur unimpeded (see Rodgers, 2006, 2007b,c). This transformation in the gang’s patterns of violence was directly linked to the gang’s involvement in the drugs trade, which constituted an activity-linked contextual factor that socialized gang members into a more violent and entrepreneurial way of being (see Rodgers, 2015).

These contextual circumstances, however, changed quite rapidly due to the evolution of drug dealing in *barrio* Luis Fanor Hernández, and more specifically its professionalization. By 2005, the local drug economy was being run by a *cartelito* which sought to suppress the *barrio* Luis Fanor Hernández gang, as a potential rival for the local monopoly over violence. In 2006, after a series of confrontations that left several gang members critically injured and one dead – executed ‘as a warning to the others’, as a member of the *cartelito* called Mayuyu put it in an interview in 2012 – the gang effectively ceased to exist as a collective unit in *barrio* Luis Fanor Hernández. Former and wannabe gang members were actively ‘de-socialized’ from becoming involved in violence by *cartelito* enforcers who would regularly patrol the neighbourhood and intimidate them.

From 2009 onwards, the *cartelito* reduced its involvement in local drug-dealing activities, refocusing instead on drug trafficking, which opened up a space for a *barrio* Luis Fanor Hernández gang ‘revival’, as the *cartelito* no longer sought to dominate the neighbourhood but rather aimed to be invisible instead. By mid-2012, a group of a dozen 14–15-year olds had organically come together, regularly hanging out on local street corners, effectively (re)occupying the sociospatial vacuum left by the *cartelito*’s withdrawal. This group’s patterns of violence were much more ad hoc and circumstantial, but rapidly became regular. In July 2012, for example, the new gang attacked a local rival gang in a nearby neighbourhood with machetes and a home-made handgun in a manner reminiscent of the gangs in the mid-1990s. Although they were repelled, with several individuals being injured – one due to the handgun exploding in his hand when he tried to shoot it – this event led to the beginning of a perception in *barrio* Luis Fanor Hernández that ‘the gang is back’, as an inhabitant called *Doña* Yolanda put it.

Interviews with members of this new gang, however, suggested that the motivation for attacking the other neighbourhood’s gang had been one individual seeking revenge for a personal slight rather than any territorial impulse, and when I tried to explain something of the neighbourhood gang history to one of the new gang members called Ronnie, he rapidly cut me off, saying ‘who gives a shit about what those old guys did?’ To this extent, the new gang’s violence was arguably underpinned by a different form of socialization to the past. More specifically, it seemed to be related to the organic emergence of a tight-knit peer group and associated adolescent practices, and can be said to have represented yet another different iteration of socialization into violence through group dynamics. The patterns of violence that this more organic form of group socialization led to were clearly much less durable than those based on the pre-existing group dynamics of the early 1990s or the more institutionalized ones of the mid-1990s, since when I returned to *barrio* Luis Fanor Hernández in 2014, the new gang had dissipated after several of the youth involved found regular employment and were no longer able to hang out with the group. This was also the case in 2016, although the lack of a new gang seemed to be largely due to increased police repression, a contextual factor that was clearly structurally equivalent to the *cartelito*’s crushing of the gang in 2006. At the same time, new forms of virtually organized collective violence were also in the process of emerging, although their dynamics seemed to relate first and foremost to a changing local sexual political economy rather than any form of group socialization (see Rodgers, 2017).

## Conclusion

This article has sought to explore the various ways in which gang members in *barrio* Luis Fanor Hernández, a poor urban neighbourhood in Managua, the capital city of Nicaragua, have assimilated norms and practices of violence over the past two decades. It has shown how different types of violence can be related to distinct forms of socialization, and traces how some of these changed over time, often for very contingent reasons, but also how there existed continuities and connections between different forms of socialization and violence. At the same time, distinctions could clearly be made between types of socialization based on specific forms of individual agency, others derived from collective group dynamics, while yet others related to broader, more contextual factors. This is important because there is a sense in which socialization processes are often considered rather monolithically. This is certainly the case of gang socialization, which is often viewed as a singular event rather than a process (see e.g. Vigil, 2002; Melde & Esbensen, 2011). The material presented above on the *barrio* Luis Fanor Hernández gang suggests instead that socialization – as Checkel (2017) argues in his introduction to this special issue – is a multilayered process that articulates together different mechanisms and process of socialization in a contingent and constantly evolving manner.

The evolutionary trajectory of the *barrio* Luis Fanor Hernández gang also illustrates how different gang member generations since 1990 dynamically experienced different configurations of socialization where individual agency, group dynamics and contextual factors interrelated in variable ways. While the latter two forms of socialization can be broken down into different iterations, with the distinction between pre-existing and institutionalized group dynamics or general, local and activity-related contextual factors both important to consider, it is striking how all tend to be systemic in their logic. This is in stark contrast with individual agency, which emerges as a highly contingent mechanism for socialization. At the same time, the latter often emerges as a socializing ‘bridge’ between different phases of the gang’s evolution, and as the means through which the most extreme forms of violence are transmitted. Having said this, it is striking how the *barrio* Luis Fanor Hernández gang’s trajectory highlights that none of these different socialization configurations institutionalized for more than a single phase. To a certain extent, this can be related to the specific nature of some of the practices of violence being socialized, including for example in relation to the knowledgeable use of firearms, which requires specific and accurate information that clearly need to be regularly re-asserted first hand across generations. In relation to contextual factors, it can also be argued that these either fostered the internalization of unsustainable norms – for example in the form of a shrinking social imaginary and individual entrepreneurial dynamics linked to drug dealing – or critical disjunctures in the local political economy – as exemplified by the rise of the *cartelito*. It is less clear why group dynamics do not necessarily institutionalize, except possibly in relation to demographic considerations of ‘critical mass’, although their sensibility to contextual factors is clearly a major element in explaining their volatility.

Understanding how and why socialization processes wax and wane, and why their effects are so volatile, is obviously a critical endeavour. In order to truly understand this issue, however, we arguably need a new research agenda, one which moves away from an organizational focus on gangs towards a closer study of the longitudinal individual life trajectories of gang members. Getting to grips with gang socialization arguably requires not just focusing on the processes and mechanisms through which norms and practices of violence are transmitted to individuals, but also a sense of how they shape their lives in the long term, including after they leave the gang. Indeed, the latter inevitably highlights the inherent volatility of socialization, as most youth who join a gang will eventually leave it through ‘natural desistance processes’ (Decker and Pyrooz, 2011: 16), and consequently generally resort less to violence (Rodgers & Jensen, 2015). How and why this takes place is not well understood, however. Does desistance signal a transformation in the way individuals absorb and process norms and practices related to violence? If this is the case, then does it indicate that socialization is primarily a group rather than an individual process, and dissipates once an individual leaves the group, or does it suggest that socialization is in fact not important in explaining violence and that other factors – such as rational choice and individual cost–benefit calculations (see Weinstein, 2007; Gates, 2017) – need to be taken into account? Or does it simply indicate the limits of socialization, suggesting for example that it is a time-bound process? Only by answering these sorts of questions can we open up the possibility for truly sustainable forms of violence reduction and peacebuilding.

## Supplementary Material

Supplementary material

## Notes

1 This name is a pseudonym, as are all the names of places and individuals mentioned in this article.

2 Having said this, the *mara* phenomenon is not simply a foreign importation by deportees, but has evolved and grown in response to domestic factors and conditions, with the majority of *mareros* no longer deportees, and *mara* gang culture more of a hybrid combining elements of US and local gang culture (Demoscopía, 2007: 49).

3 Nicaragua has no *maras* because its migration patterns are different from the latter three countries (see Rocha, 2008, for details).

4 Indeed, if Nicaragua’s official homicide statistics are to be believed, the country is one of the safest – if not the safest – countries in Central America. While there is no doubt that Nicaragua is less violent than El Salvador, Honduras and Guatemala, there is also no question that official Nicaraguan police crime statistics are hugely flawed and politically manipulated, and their extremely low levels do not accurately reflect the country’s security panorama (see Rodgers & Rocha, 2013: 49). See also Bateson (2017) for a discussion of the enabling environment for militias created by post-conflict violence and crime in Guatemala, a situation which has some parallels with Nicaragua.

5 Although female gang members are not completely unknown in Nicaragua, they are not the norm (see Rodgers, 2006: 286).

6 On the reasons for this timing, see Rodgers (2006: 278–279).

7 For an overview of methods and data collection, see this article’s Online appendix available at: https://www.prio.org/JPR/Datasets/.

8 The gang’s gender bias can be at least partly associated with the fact that being a gang member involves behaviour patterns that revolve around activities associated with *machista* ‘ideals of manhood’, such as ‘taking risk [or] displaying bravado in the face of danger’ (Lancaster, 1992: 195), and therefore inherently challenge Nicaraguan ideals of womanhood, which are associated with ‘subordination’ and ‘domestic roles, especially mothering’ (Montoya, 2003: 63). To this extent, being a gang member represents the enactment of an exaggerated form of ‘hyper-masculinity’. At the same time, relations between men and women, and notions of what it is to properly be a man or a woman, are defined not just ideologically but also through social practices. Female gang members are not unknown in Nicaragua, and the *barrio* Luis Fanor Hernández gang had a female gang member known as *la Gata* (the Cat) during the early 1990s. Although she was originally associated with the gang as a gang member’s girlfriend, *la Gata* rapidly came to be considered a fully fledged member of the gang in her own right. Her ‘femininity’ was very obviously downplayed whenever gang members talked about her, however, as they invariably described her as having been extremely ‘violent’, ‘barbaric’, or ‘fearless’, qualities that fundamentally reflect the *machismo*-inspired ideal of what a gang member should be. To this extent, she was arguably ‘masculinized’, something that further points to the absence of female roles within the gang, and implicitly suggests that *la Gata* was something of an exception.

9 Gang membership has generally been found to be a finite social role all over the world (see Covey, 2003; Hazen & Rodgers, 2014). Certainly, *barrio* Luis Fanor Hernández gang members from all epochs have often told me that ‘there is no such thing as an old gang member’, and ‘maturing out’ is considered the natural course of things, although the age at which this happens has varied in the neighbourhood over time.

10 ‘Chinese whispers’ is a game where a message is whispered from one person to another in a group, until the last player then announces the message to the entire group. Errors typically accumulate in the retellings of the message, and the version announced by the last player generally differs significantly from its original utterance.

## References

[bibr1-0022343317714299] Anderson Elijah (1999) Code of the Street: Decency, Violence, and the Moral Life of the Inner City. New York: WW Norton.

[bibr2-0022343317714299] Arendt Hannah (1969) On Violence. New York: Harcourt Brace.

[bibr3-0022343317714299] Ayling Julie (2011) Gang change and evolutionary theory. Crime, Law and Social Change 56(1): 1–26.

[bibr4-0022343317714299] Bateson Regina (2017) The socialization of civilians and militia members in the Guatemalan civil war. Journal of Peace Research 54(5): 634–647.

[bibr5-0022343317714299] Bernard Thomas (1990) Angry aggression among the ‘Truly Disadvantaged’. Criminology 28(1): 73–96.

[bibr6-0022343317714299] Cajina Roberto (2000) Nicaragua: De la Seguridad del Estado a la Inseguridad Ciudadana [From state security to citizen insecurity] In: Serbin Andrés Ferreyra Diego (eds) Gobernabilidad Democrática y Seguridad Ciudadana en Centroamérica: El caso de Nicaragua [Democratic Governance and Citizen Security in Central America: The Case of Nicaragua]. Managua: CRIES.

[bibr7-0022343317714299] Checkel Jeffrey T (2017) Socialization and violence: Introduction and framework. Journal of Peace Research 54(5): 592–605. 10.1177/0022343317714299 PMC6187852 30369635

[bibr8-0022343317714299] Covey Herbert (2003) Street Gangs Throughout the World. Springfield, IL: Charles C. Thomas.

[bibr9-0022343317714299] Curry G. David Decker Scott Pyrooz David (2014) Confronting Gangs: Crime and Community, 3rd edition Oxford: Oxford University Press.

[bibr10-0022343317714299] Decker Scott Pyrooz David (2011) Leaving the gang: Logging off and moving on. Paper commissioned by Google Ideas and the Council on Foreign Relations (CFP) (http://www.cfr.org/content/publications/attachments/SAVE_paper_Decker_Pyrooz.pdf).

[bibr11-0022343317714299] Decker Scott Winkle Barrik van (1996) Life in the Gang: Family, Friends, and Violence. Cambridge: Cambridge University Press.

[bibr12-0022343317714299] Demoscopía (2007) Maras y Pandillas, Comunidad y Policía en Centroamérica [Gangs, Community, and Police in Central America]. San José: Demoscopía.

[bibr13-0022343317714299] Fischer Claude (1975) Toward a subcultural theory of urbanism. American Journal of Sociology 80(6): 1319–1330.

[bibr14-0022343317714299] Gates Scott G (2017) Membership matters: Coerced recruits and rebel allegiance. Journal of Peace Research 54(5): 674–686.

[bibr15-0022343317714299] Hagedorn John (2008) A World of Gangs: Armed Young Men and Gangsta Culture. Minneapolis, MN: University of Minnesota Press.

[bibr16-0022343317714299] Hazen Jennifer Rodgers Dennis, eds (2014) Global Gangs: Street Violence across the World. Minneapolis, MN: University of Minnesota Press

[bibr17-0022343317714299] Jensen Steffen (2008) Gangs, Politics and Dignity in Cape Town. Chicago, IL: University of Chicago Press.

[bibr18-0022343317714299] Keiser, R Lincoln (1969) The Vice Lords: Warriors of the Streets. New York: Holt, Rinehart & Winston.

[bibr19-0022343317714299] Klein Malcolm Maxson Cheryl (2006) Street Gang Patterns and Policies. New York: Oxford University Press.

[bibr20-0022343317714299] Kruijt Dirk Koonings Kees (1999) Societies of Fear: The Legacy of Civil War, Violence and Terror in Latin America. London: Zed.

[bibr21-0022343317714299] Lancaster Roger (1992) Life Is Hard: Machismo, Danger, and the Intimacy of Power in Nicaragua. Berkeley, CA: University of California Press.

[bibr22-0022343317714299] Lepoutre David (1997) Cœur de Banlieue: Codes, Rites et Langages [The Heart of the Suburbs: Codes, Rituals, and Language]. Paris: Odile Jacob.

[bibr23-0022343317714299] Manwaring Max (2005) Street Gangs: The New Urban Insurgency. Carlisle, PA: US Army War College.

[bibr24-0022343317714299] Melde Chris Esbensen Finn-Aage (2011) Gang membership as a turning point in the life course. Criminology 49(2): 513–552.

[bibr25-0022343317714299] Montoya Rosario (2003) House, street, collective: Revolutionary geographies and gender transformation in Nicaragua, 1979–99. Latin American Research Review 38(2): 61–93.

[bibr26-0022343317714299] Núñez Juan Carlos (1996) De la Ciudad al Barrio: Redes y Tejidos Urbanos en Guatemala, El Salvador y Nicaragua [From city to neighborhood: Urban networks and fabric in Guatemala, El Salvador and Nicaragua]. Guatemala City: Universidad Rafael Landívar.

[bibr27-0022343317714299] Orozco Roberto (2012) IV Encuesta sobre Percepción de la Seguridad Ciudadana [IV survey on citizen security perceptions]. Managua: IEEPP.

[bibr28-0022343317714299] Rocha José Luis (2000a) Pandilleros: La mano que empuña el mortero [Youth Gang Members: The Hand that Rocks the Mortar Launcher]. Envío 216: 17–25.

[bibr29-0022343317714299] Rocha José Luis (2000b) Pandillas: Una cárcel cultural [Youth Gangs: A Cultural Prison]. Envío 219: 13–22.

[bibr30-0022343317714299] Rocha José Luis (2007) Lanzando piedras, fumando ‘piedras’: Evolución de las pandillas en Nicaragua 1997–2006 [Throwing stones, smoking ‘stones’: The evolution of gangs in Nicaragua 1997–2006]. Cuaderno de Investigación No. 23 Managua: UCA.

[bibr31-0022343317714299] Rocha José-Luis (2008) La Mara 19 tras las huellas de las pandillas políticas [The Mara 19 behind the traces of political gangs]. Envío 321: 26–31.

[bibr32-0022343317714299] Rocha José-Luis (2013) Violencia Juvenil y Orden Social en el Reparto Schick: Juventud Marginada y Relación con el Estado [Youth violence and social order in the Reparto Schick: Marginalized youth and their relations with the state]. Inter-American Development Bank discussion paper no. IDB-DP-308 Washington, DC: IADB (http://publications.iadb.org/bitstream/handle/11319/5772/IDB-DP-308_Violencia_Juvenil_y_Orden_Social_en_el_Reparto_Schick.pdf).

[bibr33-0022343317714299] Rodgers Dennis (2000) Living in the shadow of death: Violence, Pandillas, and social disintegration in contemporary urban Nicaragua. Unpublished PhD thesis. Department of Social Anthropology, University of Cambridge.

[bibr34-0022343317714299] Rodgers Dennis (2006) Living in the shadow of death: Gangs, violence, and social order in urban Nicaragua, 1996–2002. Journal of Latin American Studies 38(2): 267–292.

[bibr35-0022343317714299] Rodgers Dennis (2007a) Joining the gang and becoming a *broder*: The violence of ethnography in contemporary Nicaragua. Bulletin of Latin American Research 26(4): 444–461.

[bibr36-0022343317714299] Rodgers Dennis (2007b) When vigilantes turn bad: Gangs, violence, and social change in urban Nicaragua In: Pratten David Sen Atreyee (eds) Global Vigilantes. London: Hurst, 349–370.

[bibr37-0022343317714299] Rodgers Dennis (2007c) Managua In: Koonings Kees Kruijt Dirk (eds) Fractured Cities: Social Exclusion, Urban Violence and Contested Spaces in Latin America. London: Zed, 71–85.

[bibr38-0022343317714299] Rodgers Dennis (2014) After the gang: Pathways of de-socialization from violence in Nicaragua. Paper presented to the 2nd ‘Socialization and Organized Political Violence’ workshop, Yale University, 17–18 October.

[bibr39-0022343317714299] Rodgers Dennis (2015) The moral economy of murder: Violence, death, and social order in gangland Nicaragua In: Auyero Javier Bourgois Philippe Scheper-Hughes Nancy (eds) Violence at the Urban Margins. Oxford: Oxford University Press, 21–40.

[bibr40-0022343317714299] Rodgers Dennis (2016) Critique of urban violence: Bismarckian transformations in contemporary Nicaragua. Theory, Culture, and Society 33(7–8): 85–109.

[bibr57-0022343317714299] Rodgers Dennis (2017) Temporality and epistemological disjuncture: The perils, pitfalls, and possibilities of longitudinal ethnographic research. Paper presented to the ‘Longitudinal Ethnography of Violence’ workshop, Netherlands Institute for the Study of Crime and Law Enforcement (NSCR), Amsterdam, 22–23 June.

[bibr41-0022343317714299] Rodgers Dennis Baird Adam (2015) Understanding gangs in contemporary Latin America In: Decker Scott Pyrooz David (eds) Handbook of Gangs and Gang Responses. New York: Wiley.

[bibr42-0022343317714299] Rodgers Dennis Jensen Steffen (2015) The problem with templates: Learning from organic gang-related violence reduction. Stability: International Journal of Security and Development 4(1): 1–16.

[bibr43-0022343317714299] Rodgers Dennis Luis Rocha José (2013) Turning points: Gang evolution in Nicaragua. Small Arms Survey Yearbook 2013: Everyday Dangers. Cambridge: Cambridge University Press, 46–73.

[bibr44-0022343317714299] Sánchez Jankowski Martín (1991) Islands in the Street: Gangs and American Urban Society. Berkeley, CA: University of California Press.

[bibr45-0022343317714299] Sánchez Jankowski Martín (2003) Gangs and social change. Theoretical Criminology 7(2): 191–216.

[bibr46-0022343317714299] Stretesky Paul Pogrebin Mark (2007) Gang-related gun violence: Socialization, identity, and self. Journal of Contemporary Ethnography 36(1): 85–114.

[bibr47-0022343317714299] Suttles Gerald (1968) The Social Order of the Slum: Ethnicity and Territory in the Inner City. Chicago, IL: University of Chicago Press.

[bibr48-0022343317714299] Thrasher Frederick (1927) The Gang: A Study of 1,313 Gangs in Chicago. Chicago, IL: University of Chicago Press.

[bibr49-0022343317714299] UNODC (United Nations Office on Drugs and Crime) (2007) Crime and Development in Central America: Caught in the Crossfire. Vienna: United Nations (https://www.unodc.org/documents/data-and-analysis/Central-america-study-en.pdf).

[bibr50-0022343317714299] Valdez Al (2011) The origins of Southern California Latino gangs In: Bruneau Thomas Dammert Lucia Skinner Elizabeth (eds) Maras: Gang Violence and Security in Central America. Austin, TX: University of Texas Press.

[bibr51-0022343317714299] Vigil James Diego (1988) Barrio Gangs: Street Life and Identity in Southern California. Austin, TX: University of Texas Press.

[bibr52-0022343317714299] Vigil James Diego (2002) A Rainbow of Gangs: Street Cultures in the Mega-City. Austin, TX: University of Texas Press.

[bibr53-0022343317714299] Weegels Julienne (2017) Tracing the Nicaraguan prisoner: Moving between marginality, violence, and change. Unpublished PhD thesis, Department of Anthropology, University of Amsterdam.

[bibr54-0022343317714299] Weinstein Jeremy (2007) Inside Rebellion: The Politics of Insurgent Violence. Cambridge: Cambridge University Press.

[bibr55-0022343317714299] Whyte William Foote (1943) Street Corner Society: The Structure of an Italian Slum. Chicago, IL: University of Chicago Press.

[bibr56-0022343317714299] Yablonsky Lewis (1963) The Violent Gang. New York: Macmillan.

