# Supplementary material for: Bróderes in arms: Gangs and the socialization of violence in Nicaragua
Source: J Peace Res. 2017 Sep 12;54(5):648–60. doi: 10.1177/0022343317714299 (PMC6187852; doi:10.1177/0022343317714299)
Supplement: Supplementary material [file Rodgers_-_Final_Online_appendix.docx]

***Bróderes* in arms: Gangs and the socialization of violence in Nicaragua**

**Online appendix:**

**Methods and data collection**

The empirical material presented below is drawn from ongoing longitudinal ethnographic research that began in July 1996, when I first went to Nicaragua to carry out a year’s fieldwork for my social anthropology doctorate (Rodgers, 2000). I did not go to Nicaragua to study gangs; my focus on the phenomenon was in fact in many ways largely accidental, contingent on the fact that during my first couple of months in Nicaragua I suffered several violent encounters with gangs and then subsequently moved into a neighbourhood – *barrio* Luis Fanor Hernández – that happened to have a particularly notorious local gang. Both of these experiences – as well as the importance of the topic in local discourses – firmly fixed my attention on gangs as a focus of investigation and set the tone for my research. As a result of a series of perhaps somewhat unlikely events, within a few weeks of directing my investigative attentions towards gangs, I ended up being initiated into the *barrio* Luis Fanor Hernández gang. During the course of the subsequent ten months, I therefore spent significant amounts of my time hanging out with gang members on street corners and in their homes, smoking, drinking, chatting, as well as participating in a range of gang activities, both violent and non-violent (see Rodgers, 2007, for further details about my research, including in particular with regard to ethical considerations).^[[1]](#footnote-1)^

This classic method of ‘participant observation’ – the mainstay of anthropological research (see Agar, 1996) – allowed me to rapidly familiarise myself with gang norms, codes, and behaviours, and it gave me extensive access to gang members, also allowing for open and frank interviews and exchanges that were not clouded by fear or mistrust (on either side). I was able to hear from gang members what it was that had motivated them to join the gang, how they perceived themselves, as well as obtain extensive details about their illegal activities. I was able to compare their discourses against their everyday practices, as well as observe individuals acting in a range of different circumstances, including some that would normally have been impossible for a non-gang member to observe. More generally, I engaged in what Wacquant (2004: viii) has termed ‘carnal ethnography’, experiencing – obviously only up to a point, within the limits of my particular standpoint as a foreigner and an anthropologist^[[2]](#footnote-2)^ – a ‘moral and sensual conversion to the cosmos under investigation’ that allowed me to better understand the norms and practices of the gang, as well as the motivations and behaviours of individual members, including in relation to their violence. As a gang member I was for example called upon to participate in the defence of the neighbourhood against the incursions of enemy gangs, and was therefore able to experience first hand and highly viscerally the nature and logic of gang warfare (see Rodgers, 2007).

The fact that I joined the gang also provided the foundation for my longitudinal research. Although I formally ‘retired’ from the gang when I left Nicaragua in July 1997, and the gang had changed significantly when I returned to *barrio* Luis Fanor Hernández for three months in 2002, I was trusted as an ‘old timer’, and both new and old gang members were willing to share details about their illegal activities, including about the drugs trade, whenever I sought them out to chat and find out how their lives had changed since my last visit. This continued to be the case during all my subsequent visits to the neighbourhood in 2003, 2007, 2009, 2012, 2014, and 2016, despite gang member turnover. At the same time, while I continue to stay in *barrio* Luis Fanor Hernández every time I visit Nicaragua, and I still spend significant amounts of time hanging out on street corners with both current and former gang members, as my research has progressed, I have spent less time carrying out participant observation – particularly compared to my first two visits in 1996-1997 and 2002 – and have increasingly focused on carrying out more purposeful one-on-one in-depth interviews with former and current gang members. This is partly due to the shorter durations of these subsequent return visits – which have generally lasted one to two months – but it is also because as time has gone by I have become ever more intimately familiar with both the *barrio* Luis Fanor Hernández context and gang, and have therefore been able to engage in more targeted investigations (see Rodgers, 2014a, for a further details about the benefits and disadvantages relating to this evolution in my research approach, as well as the way that its longitudinal nature has shaped the kinds of evidence I’ve gathered and also my reporting of findings over the years).

In particular, while over the course of the past two decades of research I have sought to have some form of direct interaction with every single individual who has been a member of the *barrio* Luis Fanor Hernández gang since 1996, I have also carried out formal, one-off interviews with about half of the total number.^[[3]](#footnote-3)^ This subset of 70 individuals was selected through a combination of serendipity, convenience, and purposeful sampling, and is generally representative of the population of gang members that have emerged in *barrio* Luis Fanor Hernández since 1996. From my second visit in 2002 onwards, however, I have also engaged in regularly ‘repeat interviewing’ a second, smaller subset of 17 gang members. I began with an initial group of seven gang members whom I first interviewed in the 1996-97, adding two more individuals to this longitudinal sample in 2002, two more in 2003, another two in 2007, three more in 2009, and one more in 2012, to reflect evolving gang member generations. I interview the individuals in this group every time I have returned to *barrio* Luis Fanor Hernández, although in four cases there have been gaps in my interviewing, due to one individual temporarily migrating to Costa Rica, and three others being incarcerated (one twice). On the basis of my broader contextual knowledge, I believe that these 17 individuals offer a set of “archetypal” *barrio* Luis Fanor Hernández gang member trajectories that are particularly valuable in providing a dynamic picture of the gang’s evolving social practices, as well as permitting the exploration of the long term consequences of gang membership across different iterations of the *barrio* Luis Fanor Hernández gang (see Rodgers, 2014b).^[[4]](#footnote-4)^

Beyond chronicling individual life courses in as detailed a manner as possible in the case of the latter, my interviewing of gang members has not followed any set ‘protocols’ beyond generally discussing the local gang and its dynamics, the perceived social consequences of the gang phenomenon, and elucidating individuals’ phenomenological experience of it. Generally, ethnographic research can be said to aim to collect ‘theoretically representative’ – as opposed to a ‘statistically representative’ – data,^[[5]](#footnote-5)^ partly due to the fact that it is rarely replicable, because it is extremely contingent and highly dependent on the ethnographer’s particular skills set (although this is arguably true – but not generally acknowledged – of all research involving the collection of primary data). At the same time, however, among the great strengths of ethnographic research – especially when carried out longitudinally – is that it allows for the collection of particularly fine-grained and often difficult to uncover empirical material that frequently cannot be obtained through other research methods, and also due to its participatory nature, it explicitly allows for a contextually-validated apprehension of causal mechanisms and processes that offer greater analytical insight than the simple correlation of phenomena observed independently and in isolation.

***References***

Agar, Michael (1996) *The Professional Stranger: An Informal Introduction to Ethnography*, 2^nd^ edition. New York: Academic Press.

Rodgers, Dennis (2000) *Living in the Shadow of Death: Violence,* Pandillas*, and Social Disintegration in Contemporary Urban Nicaragua*. Unpublished PhD thesis. Department of Social Anthropology, University of Cambridge.

Rodgers, Dennis (2007a) Joining the gang and becoming a *broder*: The violence of ethnography in contemporary Nicaragua. *Bulletin of Latin American Research* 26(4): 444–61.

Rodgers, Dennis (2014a) From ‘broder’ to ‘don’: Methodological reflections on longitudinal gang research in Nicaragua, 1996-2014. Keynote presentation to the FSW50 conference on ‘Anthropologists at Work: Challenges and dilemmas of qualitative fieldwork methodologies in sensitive settings’, Utrecht University, 3 July.

Rodgers, Dennis (2014b) After the gang: Pathways of de-socialization from violence in Nicaragua. Paper presented to the 2^nd^ ‘Socialization and Organized Political Violence’ workshop, Yale University, 17-18 October.

Belmonte, Thomas (1979) *The Broken Fountain*. New York: Columbia University Press.

Johnson, Jeffrey (1990) *Selecting Ethnographic Informants*. Newbury Park: Sage.

Wacquant, Loïc (2004) *Body and Soul: Notebooks of an Apprentice Boxer*. Oxford: Oxford University Press.

1. It is important to note that gang members knew that I was carrying out research about them. [↑](#footnote-ref-1)
2. There is obviously a gendered aspect to my research, insofar that I am a male researcher investigating a phenomenon that is itself extremely gendered, and this clearly played a critical role in terms of determining the research possibilities open to me. Although female gang members are not unknown in Nicaragua, all those in *barrio* Luis Fanor Hernández were young men, and many of their social practices and behaviour patterns were intimately related to *machismo*. I do not think I would have been able to have the same form of engagement with the gang that I did had I been a female researcher. On the other hand, my gender – as well as my association with the gang – also negatively impacted on the possibility of my being able to interact in a systematic manner with gang members’ girlfriends, due to *machismo*-related notions of jealousy, for example. [↑](#footnote-ref-2)
3. I have also carried out eleven formal group interviews, as well as several individual interviews with former gang members from before 1996. To these should also be added hundreds of hours of informal individual and group conversations and interactions, as well as over one hundred interviews with non-gang member inhabitants of *barrio* Luis Fanor Hernández, which have almost always included some discussion of gangs. [↑](#footnote-ref-3)
4. The notion of an “archetypal” trajectory is (more or less) inspired by Belmonte’s (1979) classic study of the Neapolitan criminal underworld during the late 1960s and early 1970s, in which he drew on the Jungian notion of an “archetype” – the idea that there exist basic cognitive models through which the “collective unconscious” understands the world – in order to conceptualize how social life in the Neapolitan underworld was underpinned by a limited repository of “protean” expressive forms of being, such as the “trickster” or the “scapegoat”, for example. Belmonte’s study offers a series of detailed portraits of individuals corresponding to different archetypes, in order to get to grips with the broader underlying dynamics of the world these individuals live in. As such, the notion of an archetype is very much illustrative rather than representative, but it arguably constitutes a particularly useful heuristic trope through which to think about situations where a group of individuals’ lives tends to conform to a limited number of different trajectories. [↑](#footnote-ref-4)
5. On this epistemo-methodological issue, see Johnson (1990). [↑](#footnote-ref-5)
